# A Novel Genetic Screen Implicates Elm1 in the Inactivation of the Yeast Transcription Factor SBF

**DOI:** 10.1371/journal.pone.0001500

**Published:** 2008-01-30

**Authors:** Emily N. Manderson, Mohan Malleshaiah, Stephen W. Michnick

**Affiliations:** Département de Biochimie, Université de Montréal, Montreal, Quebec, Canada; University of Minnesota, United States of America

## Abstract

**Background:**

Despite extensive large scale analyses of expression and protein-protein interactions (PPI) in the model organism *Saccharomyces cerevisiae*, over a thousand yeast genes remain uncharacterized. We have developed a novel strategy in yeast that directly combines genetics with proteomics in the same screen to assign function to proteins based on the observation of genetic perturbations of sentinel protein interactions (GePPI). As proof of principle of the GePPI screen, we applied it to identify proteins involved in the regulation of an important yeast cell cycle transcription factor, SBF that activates gene expression during G1 and S phase.

**Methodology/Principle Findings:**

The principle of GePPI is that if a protein is involved in a pathway of interest, deletion of the corresponding gene will result in perturbation of sentinel PPIs that report on the activity of the pathway. We created a fluorescent protein-fragment complementation assay (PCA) to detect the interaction between Cdc28 and Swi4, which leads to the inactivation of SBF. The PCA signal was quantified by microscopy and image analysis in deletion strains corresponding to 25 candidate genes that are periodically expressed during the cell cycle and are substrates of Cdc28. We showed that the serine-threonine kinase Elm1 plays a role in the inactivation of SBF and that phosphorylation of Elm1 by Cdc28 may be a mechanism to inactivate Elm1 upon completion of mitosis.

**Conclusions/Significance:**

Our findings demonstrate that GePPI is an effective strategy to directly link proteins of known or unknown function to a specific biological pathway of interest. The ease in generating PCA assays for any protein interaction and the availability of the yeast deletion strain collection allows GePPI to be applied to any cellular network. In addition, the high degree of conservation between yeast and mammalian proteins and pathways suggest GePPI could be used to generate insight into human disease.

## Introduction

Progress in large-scale experimental strategies in the last decade is creating the framework for a molecular theory of the cell: from the structure of the genome to the principles that organize biochemical and gene regulatory networks. Genetic and proteomic methodologies are commonly applied in the model organism *Saccharomyces cerevisiae* to decode genomic sequence information into a meaningful understanding of protein function. For example, genetic screening methods including synthetic genetic array analysis, diploid synthetic lethality analysis by microarray, and epistatic miniarray profiles, imply functional links between proteins by identifying pairs of mutations in non-allelic genes that cause aggravating or alleviating effects on growth [1], [2], [3], [4]. However, the ability to demonstrate interactions between two genes is restricted to those that result in a measurable change in phenotype, such as fitness.

To maximize the knowledge that can be obtained from large-scale genomic and proteomic experiments, focus is now being placed on systems biology approaches in which datasets are combined to learn more information than can be gathered from any one dataset alone. Recently, combining genetic interactions with protein-protein interaction (PPI) data has been shown to generate valuable insight into relationships between protein complexes and genetically defined epistasis groups [1]. In addition, functional protein complex dynamics have been inferred from comparison of PPI data with gene expression co-variation for intrinsically dynamic processes, including replicative and respiratory cell cycles where timing of protein complex assembly and gene expression are assumed to be tightly linked [5], [6]. In these efforts, PPI data are used as a tool of inference, whereas here we show how dynamic PPIs can be used as direct and general sensors of the activity of any cellular pathway to provide mechanistic insights into the roles of proteins in a cellular process.

**Figure 1:**
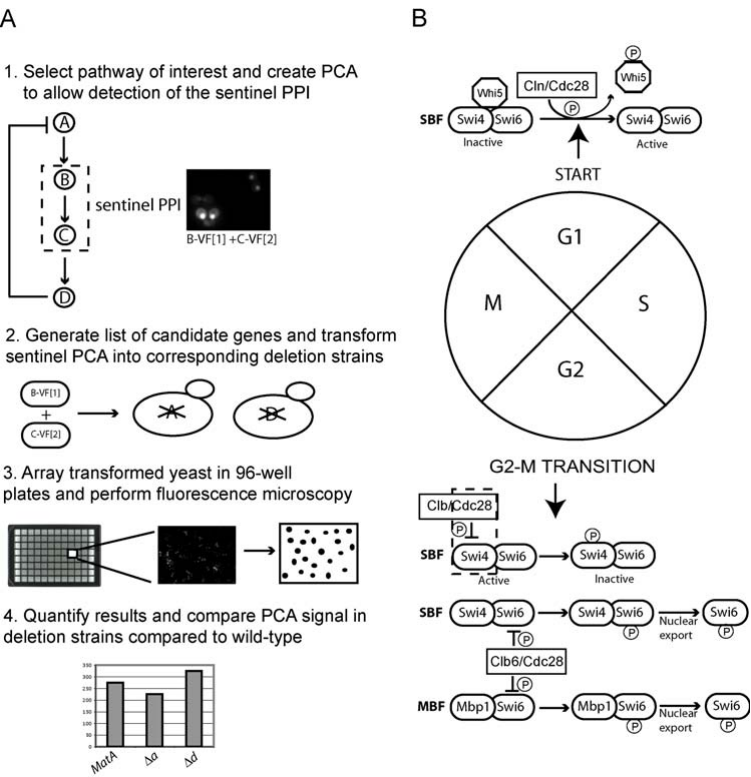
The GePPI screening strategy to identify proteins involved in the inactivation of SBF via phosphorylation by Clb/Cdc28. (A) A schematic representation of the GePPI screening strategy. 1) A biological pathway of interest is selected and a PCA assay is created that detects one or more sentinel PPIs of this pathway in wild-type yeast. In this example, protein A activates the sentinel interaction between proteins B and C, whereas protein D inhibits the interaction via a negative feedback loop. 2) Candidate genes are selected and plasmids encoding the PCA fusion proteins for each assay are transformed into the corresponding deletion strains. 3) Transformed deletion strains are screened in 96-well plates by fluorescence microscopy and images are collected and processed using image analysis software. 4) Strains are selected for which the PCA signal is significantly decreased or increased, as this type of analysis can be easily automated for yeast without the use of counter-stains that are required to identify changes in sub-cellular localization. In this example, deletion of protein A results in a decrease in the PCA signal, whereas deletion of protein D results in an increase in signal. (B) Regulation of SBF and MBF throughout the cell cycle. Activation of SBF involves phosphorylation of the SBF-associated repressor Whi5 by Cln/Cdc28 during G1. Phosphorylation of Whi5 leads to its dissociation from SBF followed by nuclear export. Inactivation of SBF in G2/M involves phosphorylation of Swi4 by Clb/Cdc28 activity. Regulation of MBF is less well understood but phosphorylation of Swi6 by Clb6/Cdc28 followed by Swi6 nuclear export may be a mechanism of inactivation of both SBF and MBF. The sentinel interaction between Cdc28 and Swi4 is indicated by dashed box.

We present a novel screening strategy in which genetics and proteomics are incorporated to detect genetic perturbations of protein interactions (GePPI) in order to assign function to previously uncharacterized or characterized proteins (Figure 1A). The principle is that if a protein encoded by a candidate gene plays a role in a biological pathway of interest, deletion of the gene will result in perturbation of a sentinel PPI within the pathway. The protein can be implicated in any step in the pathway upstream of the interaction measured, wherein the change propagates through the pathway resulting in a perturbation of the PPI. Alternatively, a protein can be involved in a downstream positive or negative feedback event that regulates the sentinel PPI. The sentinel PPI is detected using protein-fragment complementation assays (PCA) (Reviewed in [7]), and perturbations are measured by fluorescence microscopy and image analysis of the PCA in selected yeast deletion strains. We previously showed that fluorescent PCAs can detect spatial and/or temporal perturbations of PPIs *in vivo* in mammalian cells, following addition of drugs, siRNAs, or hormones [8], [9], [10], [11], [12]. Perturbations of the sentinel PPI could be due to a number of different processes including, removal of a mediator or inhibitor of the interaction, changes in the rate of protein synthesis or degradation, changes in protein localization, or post-translational modifications.

To illustrate feasibility of the GePPI screen, we applied it to the discovery of mechanisms underlying regulation of the yeast cell cycle. A key aspect of this regulation involves proper timing of activation and inactivation of transcription factors by the cyclin dependent kinase, Cdc28. Cdc28 is activated by three G1-specific cyclins, Cln1-3 and six mitotic B-type cyclins, Clb1-6. Two heterodimeric transcription factors, SBF and MBF activate G1/S-phase gene expression and each contains a common transactivation protein, Swi6 and a unique DNA binding protein, Swi4 and Mbp1 respectively (Figure 1B). SBF is activated in G1 by Cln/Cdc28 indirectly via phosphorylation of the SBF repressor Whi5 [13]. SBF is later inactivated at the G2/M transition by Clb/Cdc28-dependent phosphorylation of Swi4 and Clb1 and Clb2 are the principle cyclins responsible for this inactivation [14], [15] (Figure 1B). Similar mechanisms governing regulation of MBF have not been elucidated; however, phosphorylation of Swi6 by Clb6/Cdc28 leads to nuclear export during M-phase and may contribute to inactivation of both transcription factors [16].

The interaction between Cdc28 and Swi4 was chosen as the sentinel PPI in our GePPI screen since it represents the cell cycle regulated event of SBF inactivation. We screened the Cdc28-Swi4 PCA in 25 candidate deletion strains and showed that the serine/threonine kinase Elm1 is important in the inactivation of SBF. In addition, we present data that suggest that phosphorylation of Elm1 by Cdc28 is an important negative feedback event leading to degradation of Elm1 upon completion of mitosis.

## Results and Discussion

**Figure 2:**
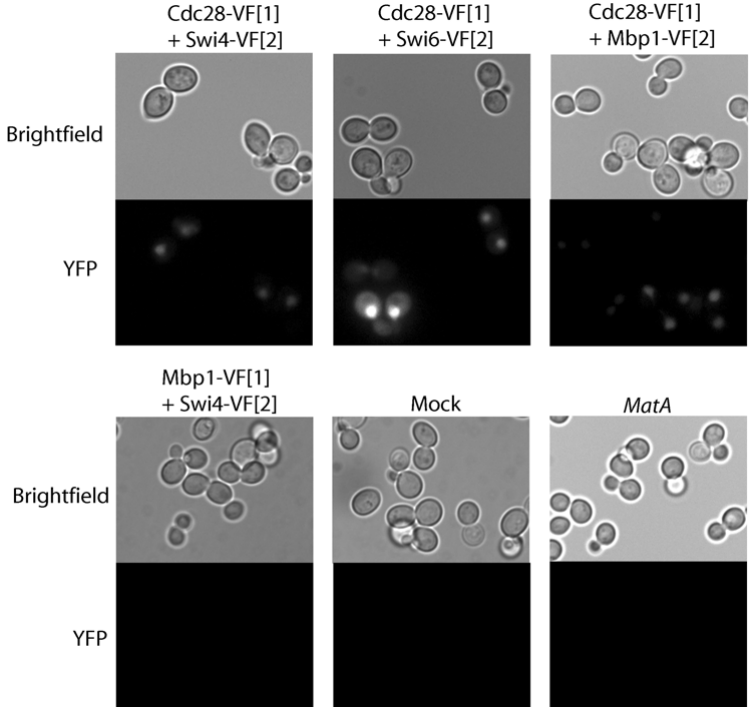
Interactions between Cdc28 and the components of SBF and MBF. Representative images of wild-type yeast transformed with the Cdc28-Swi4, Cdc28-Swi6 and Cdc28-Mbp1 yeast enhanced monomeric Venus (yEmVenus) PCAs. Fluorescence is detected in the nucleus for all three interactions. No fluorescence is detected in cells transformed with the negative control PCA Swi4-Mbp1, mock cells transformed with empty plasmids, or untransformed *MatA* cells.

In order to detect and localize PPIs in yeast, we adapted the enhanced yellow fluorescent protein ‘Venus’ PCA [11], [12] for use in *S. cerevisiae*. With the yeast enhanced Venus PCA, we observed the interaction between Cdc28 and Swi4 in the nuclei of dividing cells (Figure 2). A direct physical interaction between Cdc28 and Swi4 has not been previously reported, but was implied by the finding that Swi4 co-immunoprecipitates with Clb2 even in the absence of Swi6, and is phosphorylated by Clb2/Cdc28, whereas Swi6 is not [14], [15]. We also established assays and detected nuclear interactions of Cdc28 with Swi6 and Mbp1 *in vivo* (Figure 2). These PCAs were included as controls in the GePPI screen based on the assumption that a perturbation specific to the interaction between Clb/Cdc28 and Swi4 may or may not result in a similar perturbation of the Cdc28-Swi6 PCA since Swi4 and Swi6 form a complex. In contrast, the Cdc28-Mbp1 PCA should not be perturbed since Clb1-4 are not required for suppression of MBF activity and Mbp1 does not co-immunoprecipitate with Clb2, suggesting MBF is inactivated through an alternate mechanism [14], [15].

In order to increase the efficiency of the GePPI screen, we took a targeted approach to select candidate genes with an increased likelihood of being involved in the inactivation of SBF based on previous studies. We compared a list of 412 genes that are periodically expressed during the cell cycle with no PPI of significant reliability according to de Lichtenberg *et al* [5] to a list of 181 Cdc28 substrates identified *in vitro* [17]. Our rationale was that one or more of these genes may function upstream of the inactivation of SBF by Clb/Cdc28, and may, via phosphorylation by Cdc28 mediate positive or negative feedback regulation of Cdc28 activity. Merging of the two datasets generated a list of 25 non-essential proteins for which the deletion strain was available, to which we added the three components of SBF and MBF: Swi4, Swi6, and Mbp1; as well as the principle cyclins involved in activation and inactivation of SBF, Cln3 and Clb2 respectively (Table S1).

**Figure 3:**
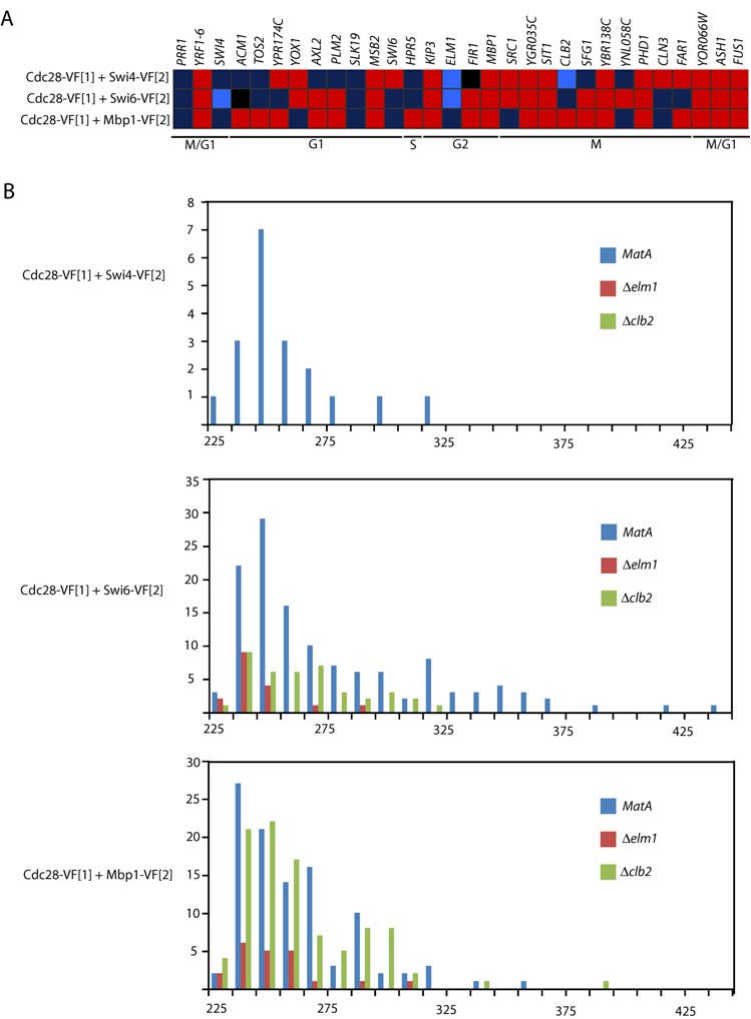
The GePPI screen identifies Elm1 as involved in the regulation of SBF by Cdc28. (A) Changes in average mean pixel intensity for the three PCAs: Cdc28-Swi4, Cdc28-Swi6 and Cdc28-Mbp1, in the 30 deletion strains. Dark red indicates increase in PCA signal, dark blue indicates decrease in PCA signal, and black represents no change in PCA signal in a deletion strain relative to *MatA*. Light blue indicates significant decreases in signal (*P*<0.0017). Genes are ordered according to time of peak expression during the cell cycle according to de Lichtenberg *et al*. [5] and the cell cycle phase of expression is indicated. (B) Representative histograms showing cell population distributions of fluorescent intensity for the three PCAs used in this study in *Mat A*, Δ*elm1* and Δ*clb2*.

The Cdc28-Swi4 sentinel PCA, as well as the Cdc28-Swi6 and Cdc28-Mbp1 PCAs were transformed into wild-type yeast and the 30 deletion strains. Each deletion strain was assigned a number to create a “blind” assay in which all steps of the screen were performed without prior knowledge of the gene name or function. The PCA signal was quantified by fluorescence microscopy and image analysis and the differences in average mean pixel intensity of each PCA between wild-type and each deletion strain are presented in Figure 3A and Figure S1. A dramatic perturbation of the Cdc28-Swi4 sentinel PPI was observed in Δ*elm1* and Δ*clb2* in that there was no signal above the autofluorescence threshold in either strain (*P* = 0.0002). The Cdc28-Swi6 PCA signal was also significantly decreased in Δ*elm1* (*P* = 7.273e-05) but not Δ*clb2* (*P* = 0.406). The Cdc28-Mbp1 PCA was not significantly perturbed in either deletion strain. Distributions of fluorescent intensity for the three PCAs in *MatA*, *Δelm1* and *Δclb2* are shown in Figure 3B.

Western blot analysis revealed that both Cdc28-VF[1] and Swi4-VF[2] fusion proteins were detectable in Δ*elm1* indicating that in the absence of Elm1 the interaction between Cdc28 and Swi4 is inhibited (Figure S2). In contrast, the Swi4 fusion protein was not detectable in Δ*clb2*, showing that it is unstable in these cells and suggesting that cells reduce SBF activity by inducing Swi4 degradation. These results are consistent with the finding that Clb1 and Clb2 are the cyclins mainly responsible for repression of SBF regulated genes and that Clb2 is sufficient for repression of SBF-induced genes in the absence of Clb1, Clb3 and Clb4 [14].

A significant decrease in the Cdc28-Swi6 PCA signal was observed in Δ*swi4* (*P* = 1.716e-05) and both PCA fusion proteins were detected. Since there is significant overlap in the target genes of SBF and MBF [18], [19], cells may decrease the inactivation of MBF by inhibiting the phosphorylation of Swi6 by Clb6/Cdc28 which leads to nuclear export, in order to compensate for the lack of SBF.

The results of the GePPI screen indicate that Elm1 plays an important role in the inactivation of SBF via phosphorylation of Swi4 by Clb/Cdc28. Elm1 is a serine/threonine kinase implicated in cytokinesis, filamentous growth and polar bud growth based on the elongated bud morphology of the deletion strain; however, Elm1 function has not been associated with regulation of SBF [20], [21], [22], [23]. To better understand the relationship between Elm1 and SBF activity, and the role of phosphorylation of Elm1 by Cdc28, we performed subsequent analysis of *MatA*, Δ*elm1*, and *elmT551A*, a strain in which we endogenously mutated the threonine in the single Cdc28 consensus site found in Elm1 to prevent its phosphorylation.

**Figure 4:**
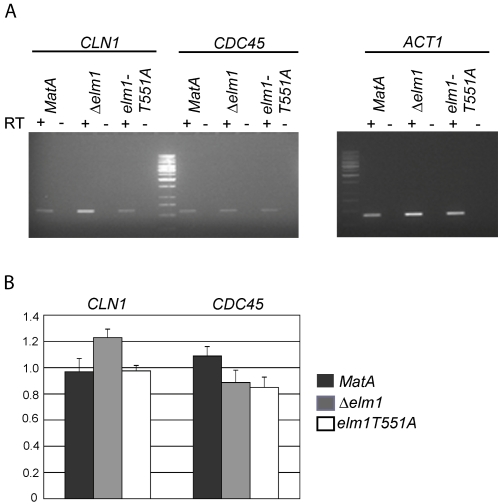
SBF activity is increased in the Elm1 deletion strain. (A) A representative result of semi-quantitative RT-PCR analysis of the SBF-specific gene, *CLN1*, the MBF-specific gene, *CDC45* and control gene, *ACT1* in *MatA*, *Δelm1* and *elm1T551A*. RT = reverse transcriptase. (B) Average expression of *CLN1* and *CDC45* normalized to *ACT1* expression, over three different experiments. Error bars represent standard deviation.

Decreased interaction between Cdc28 and Swi4 in Δ*elm1* implies diminished inactivation of SBF. In support of this hypothesis, we observed increased mRNA levels of the SBF-specific gene, *CLN1* in Δ*elm1* (*P* = 0.0118), but not the MBF-specific gene, *CDC45* (Figure 4 A,B). There was no significant increase in *CLN1* transcripts in *elm1T551A* in comparison to wild-type cells (*P* = 0.4228) (Figure 4 A,B), consistent with the finding that the Cdc28-Swi4 PCA is not perturbed in the mutant strain (Figure S3). SBF-specific targets are enriched in genes encoding proteins responsible for cell morphogenesis and budding [18], [19]. Thus, it is possible that the elongated bud phenotype of Δ*elm1* is at least partially due to a failure to inactivate SBF activated transcription of genes that induce budding.

**Figure 5:**
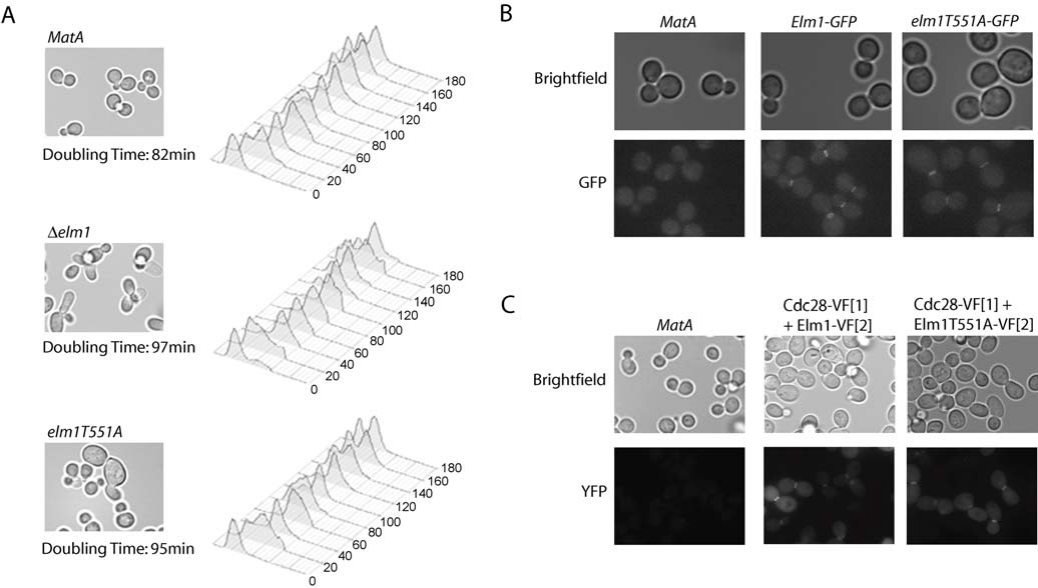
Phenotypic characterization of the Elm1 deletion strain and the elm1T551A mutant strain. (A) Morphology, doubling time and DNA replication of the three strains, *MatA*, Δ*elm1*, and *elm1T551A*. (B) Elm1-GFP and elm1T551A-GFP expressed from the endogenous *ELM1* promoter localize to the bud neck of dividing yeast cells. (C) Interaction between Cdc28 and both Elm1 and elm1T551A was detected at the bud neck of dividing yeast using yEmVenus PCA.

To further dissect the function of Elm1 and gain insight into the role of phosphorylation of Elm1 by Cdc28, we compared the phenotype of *elm1T551A* with that of Δ*elm1* and wild-type cells. The deletion strain displayed elongated buds, while in contrast a small population of *elm1T551A* cells displayed an enlarged cell phenotype, indicating that phosphorylation of threonine-551 is not required for cytokinesis but may be required for timely progression through G1 (Figure 5A). Both strains exhibited increased doubling time in comparison to wild-type cells (Figure 5A). Flow cytometry of synchronized cells showed that a small population of Δ*elm1* cells remained blocked in G2 (Figure 5A), consistent with the finding that cells lacking Elm1 undergo a prolonged mitotic delay [22]. *elm1T551A* displayed a profile of DNA replication similar to wild-type cells; however, there was an increase in the proportion of cells remaining in G1 at 180 minutes after release, which can explain its increased doubling time.

Elm1 was shown to localize to the bud neck of dividing yeast when expressed from its own promoter [23]. We tagged both Elm1 and elm1T551A at their C-termini with full-length green fluorescent protein (GFP) and observed both fusion proteins at the bud neck of dividing yeast cells, indicating the mutation does not affect the subcellular localization of the protein (Figure 5B). Fluorescent PCA analysis demonstrated that Cdc28 interacted with both Elm1 and elm1T551A predominantly at the bud neck of dividing yeast and to a lesser extent in the cytoplasm, indicating that Cdc28 is able to interact with both the wild-type and mutant forms of the protein (Figure 5C). No physical interaction of these two proteins has been reported, although a mutation in *ELM1* was shown to be synthetically lethal with the *Cdc28C127Y* mutation that causes filamentous growth [24]. Our results are consistent with the *in vitro* phosphorylation of Elm1 by Clb2/Cdc28 [17] since Clb2 is the only cyclin that localizes to the bud neck [25].

**Figure 6:**
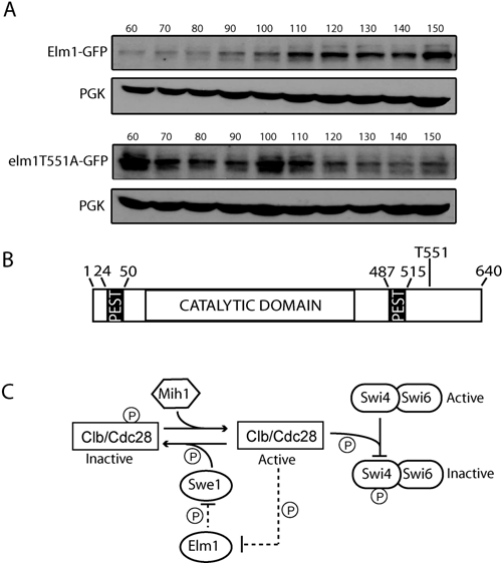
A proposed mechanism of Elm1 involvement in regulation of SBF activity in yeast. (A) Western blot analysis of Elm1-GFP and elm1T551A-GFP expressed from the endogenous promoter in synchronized cells using Anti-GFP antibody. As a loading control, blots were probed with anti-3-phosphoglycerate kinase (PGK) antibody. (B) Schematic diagram of the Elm1 protein indicating the location of the two PEST motifs in the N- and C-termini, the catalytic domain, and threonine-551 of the single full consensus site of Cdc28 phosphorylation. (C) A proposed model whereby Elm1 functions upstream of Swe1 to relieve its inhibition of Clb/Cdc28 activity. Elm1 may also be phosphorylated by Cdc28 in a negative feedback loop to induce its degradation upon completion of mitosis. Hypothetical interactions are indicated by dashed lines, solid lines represent interactions previously reported in the literature.

The majority of cells expressing elm1T551-GFP are larger than wild-type cells and those expressing Elm1-GFP, similar to the phenotype we observed for the *elm1T551A* strain, but more severe. This observation suggests that the mutation may cause a mild delay in G1, consistent with the increased doubling time and profile of DNA replication observed for *elm1T551A*. We hypothesized that phosphorylation of Elm1 by Cdc28 at threonine-551 is a signal for Elm1 degradation at the end of mitosis and in the absence of this phosphorylation signal, Elm1 levels and in turn Clb/Cdc28 activity persist, thereby inhibiting progression through the restriction point in G1. To test this hypothesis we attempted to measure the levels of the wild-type and mutant forms of Elm1 protein, but were unable to detect the proteins using an anti-Elm1 antibody. A previous study also failed to detect Elm1 by immunoblot analysis even when over-expressed from the *GAL* promoter [26]. However, the authors were able to detect an Elm1 fusion protein that was tagged with GST at its N-terminus, leading them to speculate that Elm1 normally has a short half-life and that the GST tag interfered with recognition of a PEST motif found at residues 24–50, which is proposed to serve as a signal for rapid intracellular proteolysis [26]. Similarly, we were able to detect Elm1-GFP and elm1T551A-GFP with an anti-GFP antibody in synchronized cells. We showed that Elm1-GFP was present at low levels after release from arrest in G1, and then increased steadily throughout the cell cycle. In contrast, elm1T551A-GFP was observed in the highest amount at the first time point after release from G1 arrest (Figure 6A). The combination of the *T551A* mutation and the C-terminal GFP tag may lead to stabilization of the Elm1 protein by interfering with recognition of a second PEST motif at residues 487-515 [26] (Figure 6B). This could also explain why the enlarged cell phenotype of *elm1T551A-GFP* is more severe than that of *elm1T551A*. Interestingly, a C-terminal deletion mutant of Elm1 (residues 1-420) that lacks threonine-551 displays higher kinase activity than the wild-type protein [27].

Using the GePPI screen, we identified Elm1 as a protein involved in the inactivation of SBF by Clb/Cdc28. Previous studies showed that Elm1 is required for proper timing of Clb2/Cdc28 kinase activity, that peak Elm1 protein levels correlate with maximal Clb2/Cdc28 activity, and the phenotypic consequences of knocking-out *ELM1* are much more severe in a Clb2-dependent background [22], [23]. Swe1 inhibits Clb/Cdc28 activity by phosphorylation on tyrosine-19 of Cdc28 and this inhibition seems to be specific to Clb2/Cdc28 [28]. The hyperpolarized growth and G2/M delay of *Δelm1* is suppressed by deletion of *SWE1*, or mutation of Cdc28 such that it cannot be phosphorylated on tyrosine-19 [21]. Elm1 is also required for the hyperphosphorylation of Swe1 *in vivo* at the G2/M transition [22]. Based on these findings and our results, we present a model in which Elm1 functions upstream of Swe1 to relieve its inhibitory action on Clb/Cdc28 activity, which is required for inactivation of SBF (Figure 6C). Our results also suggest that phosphorylation of Elm1 by Cdc28 is an important mechanism of inducing Elm1 degradation which in turn would lead to the suppression of Clb2/Cdc28 activity upon completion of mitosis (Figure 6C).

Using the GePPI screen we identified one of 25 (4%) candidate genes as playing a role in inactivation of SBF. Both Clb2 and Elm1 protein levels peak at the onset of mitosis, at the approximate time that Swi4 interacts with the Clb2/Cdc28 kinase [15], [23], suggesting that selecting candidates expressed in the same cell cycle phase(s) as the sentinel PPI may increase the success rate of a GePPI screen involving a cell-cycle regulated event. In contrast to large-scale screens that aim to test every gene or protein in an organism, performing a small-scale screen by selecting candidate genes based on prior knowledge can greatly increase the efficiency and reduce the cost of the screening process.

We used the GePPI screen to provide new insights into the regulation of an important cell cycle regulated event. The ability to generate PCAs with fluorescent, luminescent or simple survival-selection readouts [7] for any PPI in yeast means that one or a series of specific sensors can be created to causally link any gene to any cellular process, and to different steps in these processes. The choice of PCAs to use in such screens will be dictated by the problem being studied and specific advantages or disadvantages of the different PCAs. For instance, the PCAs based on green fluorescent protein variants reported here are useful in that they can capture qualitative perturbations such as changes in cellular locations of complexes in a gene-deleted strain. The fact that these assays are irreversible, and are therefore kinetic traps of protein complexes, means that they can capture transiently formed complexes [11], [29]. However, in some instances (but not many), trapping complexes by the folded PCA reporter protein could prevent detection, particularly of disruption of interactions. Trapping is not a general problem with PCAs and in particular, we have demonstrated that new classes of PCAs based on luciferases are completely reversible; that is, dissociation of protein complexes leads equally to unfolding and physical separation of the complementary reporter protein fragments [30], [31].

Individual PCAs combined with the availability of the deletion strain collection [32] and titratable promoter alleles [33] or hypomorphic alleles [2] to study essential genes in yeast means that GePPI could be applied to any cellular pathway for which a sentinel PPI reports on the state of the pathway in order to provide testable mechanistic hypotheses into the function of genes. The fact that over a thousand yeast genes are still listed as uncharacterized in the Saccharomyces Gene Database emphasizes the need for rapid screening strategies that can provide such insights into protein function [34]. GePPI is also complementary to screens for genetic interactions by providing a direct way to access the mechanistic origins of such interactions. The high degree of conservation of proteins and pathways between yeast and mammalian cells indicates GePPI could also be used to better understand human diseases. For example, the mammalian tumor suppressor LKB1, which is mutated in the Peutz-Jeghers familial cancer syndrome, displays heterologous function to Elm1 in yeast and therefore our results may provide insight into the mechanism of cancer development in this disease [35].

## Methods

### PCA

The enhanced yellow fluorescent protein ‘Venus’ PCA, was adapted to allow visualization of PPIs in *S. Cerevisiae*. Alanine 206 of yeast enhanced Venus (yEVenus) was mutated to lysine (a mutation that has been shown to prevent dimerization of GFP and its variants [36]) by site-directed mutagenesis of pKT103 [37], yielding yeast enhanced monomeric Venus (yEmVenus). Fragment 1, (VF[1]: amino acids 2-158) and fragment 2, (VF[2]: amino acids 159-240) of yEmVenus were amplified by PCR with the addition of a (GGGGS)_2_ linker sequence at their 5′ ends and cloned into the p413ADHcen and p415ADHcen plasmids respectively [38]. The sequences of genes of interest (without the stop codon) were amplified by PCR from genomic DNA extracted from *MatA* (BY4741) cells and cloned into the plasmids at the 5′ end of the linker to generate fusion proteins.

### Transformation of yeast with plasmids

Plasmids encoding VF[1] and VF[2] fusion proteins or empty plasmids (mock cells) were co-transformed into competent *MatA* or deletion strains (BY4741) [32]. Deletion strains were assigned a number (DS1-30) to create a “blind” assay for the measurement of PCA signal. Approximately 250 ng of each plasmid, 10 µL of competent cells, 60 µL PLATE solution (40% Polyethylene glycol 3350, 100 mM LiOAc, 10 mM Tris pH 7.5, 0.4 mM EDTA) and 8 µL of DMSO were mixed and incubated at 42°C for 20 min. Yeast were then centrifuged at 2500 RPM for 3 min, the supernatant was removed and the cells were resuspended in 500 µL of SD medium without amino acids or glucose. Approximately 20 µL of this yeast suspension were then plated on six-well plates containing SC agar, -histidine, -leucine, -lysine and incubated at 30°C for 48–72 h to obtain individual colonies. Positive colonies were verified by colony PCR.

### Homologous recombination

The stop codon of Elm1 or elm1T551A was replaced with the sequence of yeast enhanced green fluorescent protein 3 (yEGFP3) with the (GGGGS)_2_ linker at its 5′end by homologous recombination. Yeast were transformed as described above with the addition of an incubation step of the DNA, yeast and PLATE solution at room temperature for 30 min prior to the addition of DMSO and heat shock. Following heat shock, 200 µL of YPD was added to the cells, incubated at 30°C with shaking for 4 h and plated on YPD agar containing 100 µg/mL nourseothricin to select individual colonies. Positive colonies were verified by colony PCR.

### Fluorescence Microscopy and Image Analysis

Yeast strains were grown in low fluorescence medium [37] (-his, -leu, -lys) to an OD_600_ of 0.3 in order to have approximately the same number of cells per sample at the time of analysis. 70 µL of each sample were added to individual wells of a 96-well glass bottom plate (Molecular Machines) coated with Poly-L-Lysine mol wt 30,000–70,000 (Sigma P2636). Fluorescence microscropy was performed using an inverse Nikon TE 2000U microscope with 60× objective and YFP filter cube (41028, Chroma Technologies). Ten 16-bit images were captured with 750 ms exposure time for each sample with a CoolSnap HQ CCD camera (Photometrics) using Metamorph software at room temperature. Images were analyzed with a macro written in ImageJ software (NIH). PCA signal was measured by setting the threshold intensity to a minimum of 225 to exclude the autofluorescence measured for mock cells. Mean pixel intensity was measured for each particle of a minimum size of 100 pixels after the threshold was applied. The average of the mean intensity was calculated for all particles for each sample. Images of *Elm1-GFP* and *elm1T551A-GFP* were captured as described above except using a FITC filter cube (31001, Chroma Technologies).

### Elm1 mutagenesis

To mutate the threonine of the single Cdc28 consensus site (S/T-P-X-K/R) of Elm1, the coding sequence and 421 bp of 5′- and 533bp of 3′ flanking sequence were PCR amplified from genomic DNA and ligated into pRS306 [39] to generate pMNE1. The *T551A* mutation was introduced into pMNE1 by site-directed mutagenesis and the plasmid was integrated into the wild-type *ELM1* locus of BY4741 *MatA* yeast by the pop-in-pop-out strategy following digestion with BglII [40].

### RT-PCR

RNA was extracted from asynchronous yeast in logarithmic growth phase using the MasterPure Yeast RNA Purification Kit (Epicentre Biotechnologies) according to the manufacturer's protocol. Genomic DNA was digested with DNase at 37°C for 45 min. cDNA was generated from 2 µg of RNA with Ready-To-Go RT-PCR Beads (Amersham) according to manufacturer's protocol. PCR was performed on cDNA samples (including paired samples prepared after inactivation of reverse transcriptase at 95°C for 10min to serve as controls for the presence of genomic DNA) for each strain using oligos designed to amplify close to the 3′ end of each gene: *CLN1*-F: CTCAAACGCAGGTATTCAGC and *CLN1*-R: GCGATATCGAAGACGCTCTA; *CDC45*-F: TGACGATACAGATGGAGAGGA and *CDC45*-R: AGGTCAGCTTCTCCAGGAAT; *ACT1*-F: CCTACGTTGGTGATGAAGCT and *ACT1*-R: GTCAGTCAAATCTCTACCGG. PCR conditions were as follows: 95°C for 2 min, followed by 26 cycles of 95°C for 30 s, 57°C for 30 s, 72°C for 60 s, followed by 72°C for 10 min. PCR products were electrophoresed on a 1.5% agarose gel and quantified by densitometry using Quantity One software (BioRad) to determine the ratio of *CLN1* and *CDC45* transcripts in comparison to the *ACT1* control. The experiment was performed in triplicate and to normalize the data between experiments, the average of all ratios in each experiment was set to one.

### Analysis of DNA Replication by Flow Cytometry

Yeast strains were grown to an OD_600_ of 0.2 in YPD and alpha factor (Zymo Research) was added to a final concentration of 2 ug/mL and incubated at 30°C for 2 h to arrest cells in G1. Cells were washed twice and resuspended in YPD+0.1 mg/mL Pronase (Sigma) to release from arrest. 500 µL samples were collected every 20 min and fixed in EtOH. Cells were incubated with RNase solution (2 mg/mL RNase, 50 mM Tris-HCl pH 8.0) at 37°C for 1 h 45 min, followed by incubation in 200 µL 55 mM HCl and 10 mg/mL pepsin at 37°C for 30 min. Finally, cells were resuspended in 1× propidium iodide (PI) solution (180 mM NaCl, 70 mM MgCl_2_, 75 uM PI, 100 mM Tris-HCl pH 7.5) and incubated at 4°C overnight. DNA content was measured by flow cytometry using the BD LSR II System (Beckton Dickinson) and data was analyzed using FloJo Software (Treestar Inc.)

### Western blotting

To detect Elm1 and elm1T551A endogenously tagged with full-length yEGFP3, strains were grown to an OD_600_ of 0.1 in YPD and then incubated at 30°C with 2 ug/mL of alpha factor (Zymo Research) for 3 h. Cultures were washed twice and resuspended in 500 ml of fresh YPD. The first wash was considered as time zero. Every 10 minutes, 25 ml aliquots were collected and centrifuged at 4000 rpm for 3 minutes. The supernatant was removed, cells were washed with 15 ml of sterile water, centrifuged again, and pellets were immediately frozen at −80°C. Frozen pellets were thawed on ice for 15 minutes and resuspended in 150 µl of yeast extract buffer (25 mM Tris pH 7.4, 250 mM NaCl, 15 mM MgCl_2_, 15 mM EDTA, 10% glycerol, 1 mM DTT, 1mM NaN_3_, 0.1% Triton X-100 and 0.25 mM sodium vanadate, 1 mM phenylmethylsulfonyl fluoride, 5 µg/ml leupeptin , 5 µg/ml pepstatin A and 1× Complete Protease Inhibitor Cocktail, EDTA-free (Roche)). Cells were lysed by vortexing in the presence of 200 µl of acid washed glass beads. Samples were centrifuged and the supernatant was collected. Samples were migrated on 12% gels, transferred to PVDF membrane (BioRad) and probed with Anti-GFP, a mixture of two monoclonal antibodies (Roche Applied Science). As a loading control, blots were stripped and probed with yeast anti-3-phosphoglycerate kinase (PGK) monoclonal antibody (Molecular Probes).

### Statistical Analysis

Comparisons of the PCA signal intensity between wild-type yeast and candidate gene deletion strains were performed using The Mann-Whitney U test. Two-sided *P* values were calculated and the alpha level was set at 0.0017 after application of the Bonferroni correction for multiple testing (0.05/30). In order to calculate *P* values for the Cdc28-Swi4 PCA in *Δelm1* and *Δclb2*, the pixel intensity was assigned the minimum value of 225. Comparisons of RT-PCR results were performed using the unpaired T-test and one-sided *P* values were calculated with an alpha value of 0.0125 (0.05/4).

## Supporting Information

Figure S1 Magnitude of Differences in Average Mean Pixel Intensity Between MatA and Each Deletion Strain for the Three PCAs: Cdc28-Swi4, Cdc28-Swi6 and Cdc28-Mbp1.
(1.09 MB TIF)

Figure S2 Western Blot Analysis of PCA Fusion Proteins in MatA, Selected Deletion Strains and Mock Cells transformed with Empty Plasmids.
(1.94 MB TIF)

Figure S3 Comparison of Signal Between MatA and elm1T551A for the PCAs: Cdc28-Swi4, Cdc28-Swi6 and Cdc28-Mbp1.
(1.15 MB TIF)

Table S1 Candidate Genes Selected for the GePPI Screen.
(0.03 MB XLS)
