# Supplementary figures and images for: A Novel Genetic Screen Implicates Elm1 in the Inactivation of the Yeast Transcription Factor SBF
Source: PLoS One. 2008 Jan 30;3(1):e1500. doi: 10.1371/journal.pone.0001500 (PMC2198942; doi:10.1371/journal.pone.0001500)

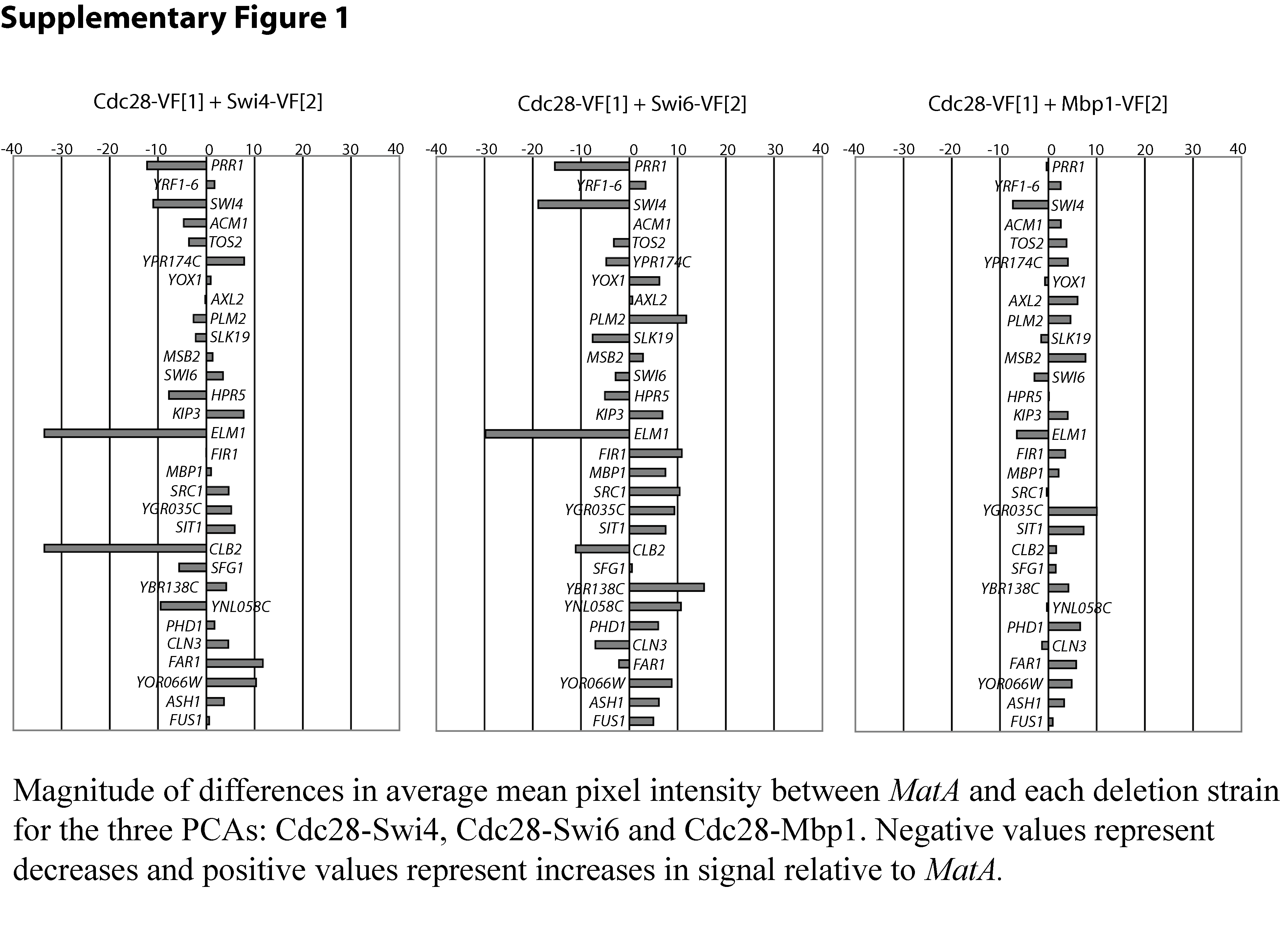

Supplement: Figure S1 — Magnitude of Differences in Average Mean Pixel Intensity Between MatA and Each Deletion Strain for the Three PCAs: Cdc28-Swi4, Cdc28-Swi6 and Cdc28-Mbp1. (1.09 MB TIF) [file pone.0001500.s001.tif]

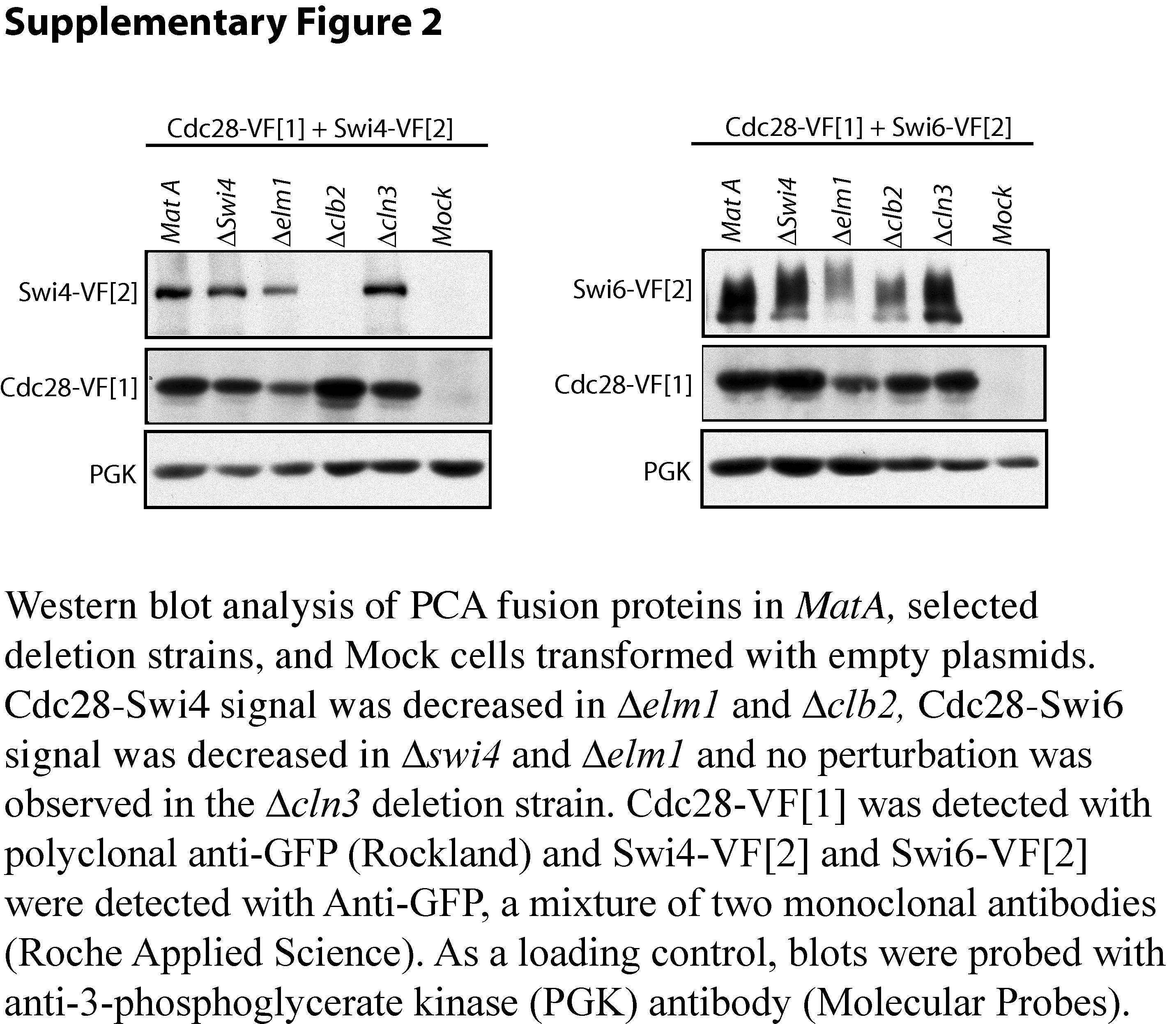

Supplement: Figure S2 — Western Blot Analysis of PCA Fusion Proteins in MatA, Selected Deletion Strains and Mock Cells transformed with Empty Plasmids. (1.94 MB TIF) [file pone.0001500.s002.tif]

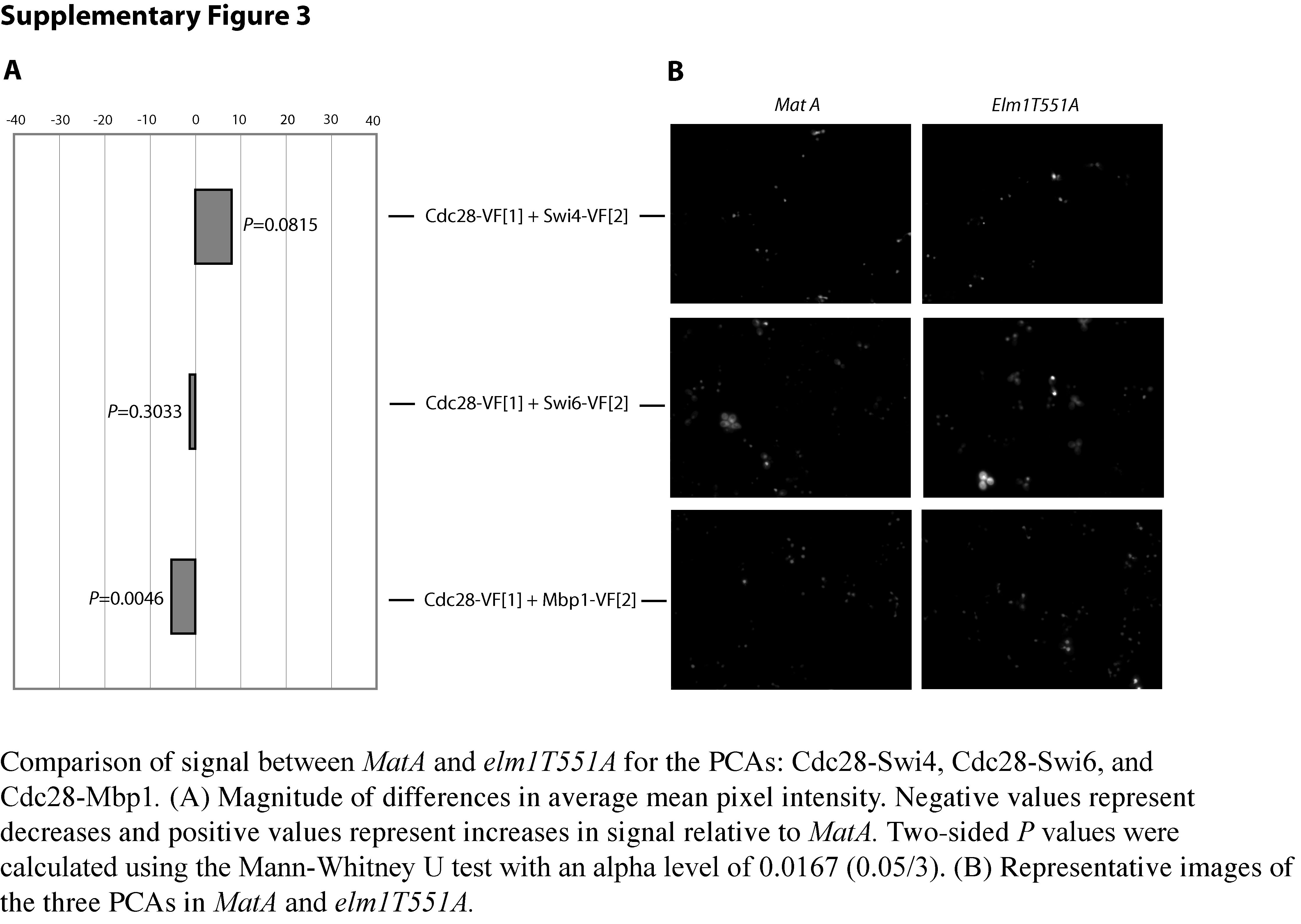

Supplement: Figure S3 — Comparison of Signal Between MatA and elm1T551A for the PCAs: Cdc28-Swi4, Cdc28-Swi6 and Cdc28-Mbp1. (1.15 MB TIF) [file pone.0001500.s003.tif]
